# Common Arterial Trunk with Interrupted Aortic Arch

**DOI:** 10.21470/1678-9741-2021-0008

**Published:** 2022

**Authors:** Lys Molina Hernandes Estephan, Aline Simões Aranda, Carlos Henrique De Marchi, Ulisses Alexandre Croti

**Affiliations:** 1 Department of Pediatrics and Pediatric Surgery, Pediatric Cardiology and Cardiovascular Surgery Service at Hospital da Criança e Maternidade de São José do Rio Preto, São Paulo, Brazil.

**Keywords:** Truncus Arteriosus, Aortic Coarctation, Cyanosis, Heart Defects, Congenital, Infant, Newborn

## Abstract

**Clinical data:** Patient diagnosed with common arterial trunk, submitted to pulmonary artery banding in another center and lost to clinical follow-up. Referred to our center at four years old, extremely cyanotic. Chest radiography: Cardiomegaly; attenuated peripheral vascular markings. Electrocardiography: Right ventricular hypertrophy. Echocardiography: Common arterial trunk, but it was not possible to analyze all the structures. Computed tomography angiography: Van Praagh type A4 common arterial trunk. Extremely hypoplastic right and left pulmonary arteries. Diagnosis: Association of aortic arch interruption type A is uncommon and should be considered. Operation: Debanding of pulmonary arteries allowing for possible future complete repair.

**Table:** 

Abbreviations, acronyms & symbols	
AA	= Ascending aorta
CAT	= Common arterial trunk
DA	= Descending aorta
LPA	= Left pulmonary artery
PDA	= Patent ductus arteriosus
RPA	= Right pulmonary artery

## CASE PRESENTATION

Female, four years old, was born in Palmas (Tocantins, Brazil), preterm birth at 33 weeks, one of triplets, with very low birth weight (1.3 kg), small for gestational age. At birth, she presented with respiratory distress and cyanosis, requiring orotracheal intubation. Echocardiogram diagnosed truncus arteriosus.

She remained in the neonatal intensive care unit for four months and was transferred to another pediatric cardiology specialized center for complete repair. Instead, she was submitted to pulmonary artery banding due to bronchodysplasia, high pulmonary vascular resistance, malnutrition, and unfavorable anatomy.

The patient lost clinical follow-up with her pediatric cardiologist and was referred to our center at four years old, with failure to thrive and neuropsychomotor developmental delay. There was also a past medical history of several hospitalizations due to respiratory distress and seizures.

Upon physical examination, the patient was in good general condition, hemodynamically stable, mean arterial pressure around 70 mmHg, and eupneic. Cyanosis, oxygen saturation around 77%, finger clubbing, and ejection systolic murmur grade 2/6 at upper left sternal border were also noted.

## TECHNICAL DESCRIPTION

### Chest Radiography

Visceral and thoracic *situs solitus* and cardiothoracic ratio of 0,57. The peripheral vascular markings were attenuated.

### Electrocardiography

Sinus rhythm, heart rate of 115 beats/min, SAQRS +150º, right bundle branch dysfunction, and right ventricular hypertrophy.

### Echocardiography

*Situs solitus* in levocardia. Normal venoatrial and atrioventricular connections and anormal ventricular arterial connection.

*Doppler* demonstrated a 15-mm ventricular septal defect with no restrictive flow, and moderate truncal valve insufficiency described as bicuspid.

It was not possible to analyze the main pulmonary artery and its branches, aortic arch, and coronaries, due to echocardiographic window technical difficulty.

For diagnostic confirmation, the patient underwent complementary computed tomography angiography.

### Computed Tomography Angiography

**Fig. 1:**
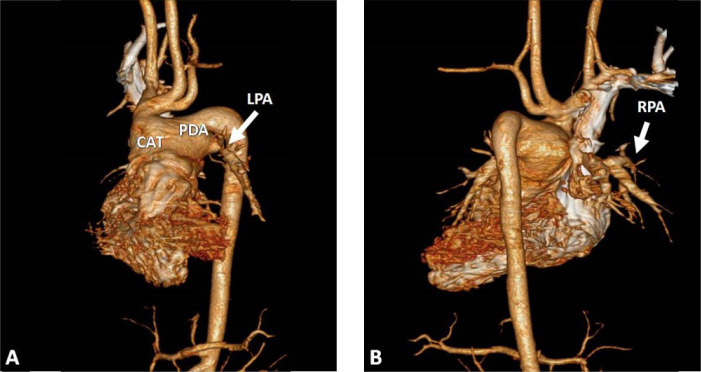
Three-dimensional volume-rendering computed tomography angiography: A) Common trunk with type A interrupted aortic arch showing a large patent ductus arteriosus (PDA) that continues to the descending aorta. Banded and very hypoplastic left pulmonary artery (LPA) (2 mm). B) Banded and very hypoplastic right pulmonary artery (RPA) (1 mm) originated from the sides of the common trunk as the LPA, with significant distance between them. CAT=common arterial trunk 1

Common trunk that trifurcates into banded and very hypoplastic right and left branch pulmonary arteries, ductal continuation to descending aorta, and ascending aorta emerging from common trunk as a side branch. Right and left pulmonary arteries originating from the sides of the common trunk with significant distance between them. Thus, confirming diagnosis of Van Praagh type A4 common arterial trunk (CAT) (Figure 1)^[1]^.

Right and left pulmonary arteries are stenotic at the origin (coinciding with the surgical bandage), measuring 1 mm on the right side and 2 mm on the left side.

## COMMENT

### Diagnosis

*Truncus arteriosus* or CAT is defined as a single vessel usually arising from both ventricles originating systemic, pulmonary, and coronary artery circulation. Significant morphological variability is found in the pattern of great arterial branching from the common trunk. The clinical presentation is dependent on morphological variation, degree of truncal valve regurgitation, and relative resistances of the pulmonary and systemic arterial vascular beds^[2]^.

From an epidemiological point of view, CAT is an uncommon congenital cardiac malformation accounting for approximately 1 to 4% of all congenital heart diseases^[2]^. The association of CAT with interruption of the aortic arch is found in approximately 11 to 19% of patients^[3]^. The interruption is classified as type A, B, or C, according to the location of discontinuity^[3]^. In a clinical study of 70 patients, 10% had interruption of the aortic arch, and only two patients had interruption distal to the left subclavian artery (type A)^[4]^.

Russel et al.^[5]^ proposed a simplified categorization for CAT, which aimed to assess whether the common trunk itself continued primarily to supply the aortic or pulmonary component. In the setting of pulmonary dominance, the patient also has hypoplastic or interrupted aortic arch.

Type A aortic arch interruption in association with CAT is relatively uncommon, has worse prognosis, and should be taken into consideration^[1]^. Also, we must consider differential diagnosis of transposition of the great arteries with interventricular communication, hypoplastic left heart syndrome, tetralogy of Fallot with pulmonary valve agenesis, and complex heart diseases without pulmonary stenosis^[6]^.

Precise diagnosis was confirmed by computed tomography angiography, however, despite correct diagnosis, the patient was referred cyanotic, with extremely hypoplastic pulmonary arteries, thus contraindicating the complete repair.

### Operation

After thorough multidisciplinary heart team discussion, the decision was debanding of right and left pulmonary artery branches. It was performed through median sternotomy and diagnosed a CAT with primary supply to the lungs (pulmonary dominance) and an interrupted aortic arch type A (distal to the left subclavian artery).

**Fig. 2:**
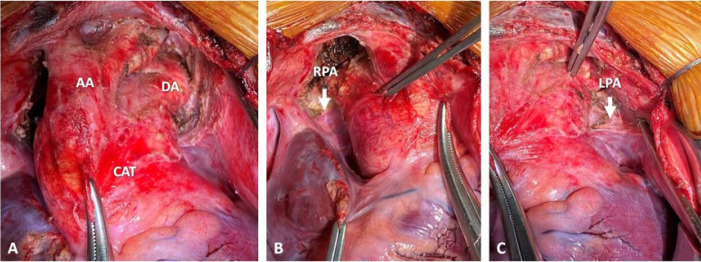
Surgical images. A) Common arterial trunk (CAT) originating the base vessels (brachiocephalic trunk, left carotid, and left subclavian artery) and ductal continuation to descending aorta (DA), configuring type A interrupted aortic arch. B) Hypoplastic right pulmonary artery (RPA) branch due to pulmonary artery banding. C) Hypoplastic left pulmonary artery (LPA) branch due to pulmonary artery banding. AA=ascending aorta

As shown in angiotomography, the right and left pulmonary branches were extremely hypoplastic. The procedure was concluded as planned, aiming the development of the pulmonary artery branches, and allowing for possible future complete repair (Figure 2). Immediately after debanding, saturation increased to 88%.

The patient had no postoperative complications and remained hospitalized for five days. She was discharged without complaints and with oxygen saturation of 93%. Currently, the patient is in clinical follow-up without use of medications; future imaging tests will be required for reintervention planning.

**Table:** 

Authors' roles & responsibilities	
LMHE	Substantial contributions to the conception or design of the work; or the acquisition, analysis, or interpretation of data for the work; drafting the work or revising it critically for important intellectual content; agreement to be accountable for all aspects of the work in ensuring that questions related to the accuracy or integrity of any part of the work are appropriately investigated and resolved; final approval of the version to be published
ASA	Substantial contributions to the conception or design of the work; or the acquisition, analysis, or interpretation of data for the work; drafting the work or revising it critically for important intellectual content; agreement to be accountable for all aspects of the work in ensuring that questions related to the accuracy or integrity of any part of the work are appropriately investigated and resolved; final approval of the version to be published
CHM	Substantial contributions to the conception or design of the work; or the acquisition, analysis, or interpretation of data for the work; drafting the work or revising it critically for important intellectual content; agreement to be accountable for all aspects of the work in ensuring that questions related to the accuracy or integrity of any part of the work are appropriately investigated and resolved; final approval of the version to be published
UAC	Substantial contributions to the conception or design of the work; or the acquisition, analysis, or interpretation of data for the work; drafting the work or revising it critically for important intellectual content; agreement to be accountable for all aspects of the work in ensuring that questions related to the accuracy or integrity of any part of the work are appropriately investigated and resolved; final approval of the version to be published
